# Filling Teeth

**Published:** 1860-08

**Authors:** H. A. Smith


### FILLING TEETH.

Read before the Cincinnati Dental Association,

BY DR. H. A. SMITH.

THERE is no operation so frequently performed upon man as that of filling the teeth—none attended with more success in the attainment of the object designed, when properly done, than this; and there is no other operation in the range of our profession which requires the same degree of judgment and skill for the thorough execution.

The object of this operation is to arrest that disease to which the teeth are most subject. This is accomplished by the removal of the carious portion of the tooth, and the subsequent restoration of the part with some indestructible material, which will afford a protection from the deleterious agents in the mouth to that portion of the tooth from which the material covering the dentine is removed.

We have referred to the large share of success attending the practice of filling teeth. But before mentioning some of the requisites for this success, we will present some of the causes of failure in many cases to accomplish the object designed by this operation. The most frequent cause of failure to render any prominent good to the teeth operated on, is owing, doubtless, to a lack of skill, or as is often the case with those who are competent, an inexcusable carelessness on the part of the operator. And it is this carelessness with those who are capable, which does so much to bring this mode of treating caries into disrepute, with many persons of considerable intelligence. Numerous are the instances in every dentist's experience, where they are told by persons that it is of no use to fill their teeth ; that they have been filled by one who was reputed to be skillful in his profession, and they are now in a worse condition than they were before he saw them. It is difficult to convince such a person that the want of success in their case was owing entirely to the careless manner in which the operation was performed, and not to the mode of practice itself. Such persons reluctantly submit to have the operation repeated, and if they do, it is with a feeling, possibly, that we are all a set of impostors, and are endeavoring to get their money without rendering an equivalent. These things are unpleasant, and every dentist owes it to his profession to do at least as well as he can.

Another practice, which we can not but deprecate, is that of raising too great expectations on the part of the patient, as to the permanency of the operation—warranting the plugs for all time to come against any possible contingency of failure. Such persons usually claim infallibility, and competent operators frequently have injustice done them by this class of dentists ; for when they discover a filling that is in an imperfect condition—no matter from what cause—they at once attempt to impress the patient with the idea that he has been victimized—that the filling never was of any value—and forthwith proceed to plug the tooth in the manner it should have been done, putting upon it with the last stroke of the plugger, their warranty and seal of perfection. What honorable man in the profession has not a supreme contempt for such as these ?

Other causes of failure may exist, which are entirely beyond the control of the dentist or the patient. Frequently, when the teeth are filled in a skillful manner, and have been preserved for a great length of time, a condition of the system will be suddenly induced, causing a vitiated condition of the fluids of the mouth, resulting in a rapid destruction of the teeth, and the same plugs which only a short time previous were perfect, have now ceased to be of any value. It may be the same state of the system which induced the first appearance of caries. The teeth possessing the same defective organization they then did, the same causes must inevitably produce like effects.

Again, neglect to properly cleanse the teeth is a frequent cause of the failure of good fillings to preserve the teeth for any considerable length of time. The retention of foreign substances in contact with the teeth is a fruitful cause of caries, and when a tooth is filled, the same cause will operate more readily upon it than at first. It is impossible to produce that nice adaptation of the filling to the edges of the cavity, so as to form the perfect continuity of the enamel in its unbroken state. And a retention of these deleterious agents in contact with the plug, will most certainly produce a carious condition of the borders of the cavity.

Frequently it is quite impossible for the operator to do himself or patient justice, owing to the many disadvantages under which he labors. The patient may be timid and sensitive to pain to a degree that the dentist is prevented from proceeding with the operation in his usual thorough manner. It is a rule that each successive step in the operation should be thorough and complete before proceeding to the next, and yet I think with all of us cases occasionally present themselves, where a departure from this practice is unavoidable, if we operate at all. The only question is whether an imperfect operation is better than none. We do not propose to discuss this, however. Other causes might be enumerated why fillings fail to be of any permanent advantage. But to you who have had any considerable experience in filling teeth, they will be readily suggested.

In order to insure any good results from filling teeth, the various steps in the operation must be as well and thoroughly performed as the circumstances of the case will admit. The pathology of the mouth, before proceeding with the operation, should receive due attention ; and if the gums or other tissues of the mouth are diseased, or the salivary or mucous secretions have not their usual characteristic properties, the prope course of treatment must be resorted to, to restore the former to a state of health and the latter to their normal condition.

A careful examination of the teeth should then be made, for frequently a correct diagnosis of caries depends entirely upon physical signs. There is an entire absence of pain, and it is only by observing a discoloration or disintegration of the enamel, that we discover the existence of dental caries. In connection with the physical signs, we may have, at an early stage of the decay, vital symptoms, and in many cases the patient can very certainly point out the teeth affected, by a feeling of discomfort produced by the action of the disease upon the tooth substance. On the other hand, patients who do not suffer any from pain in a carious tooth, can locate the disease as certainly as in the first instance. This is due to a habit of observing their teeth frequently and with care. The cultivation of this habit should be encouraged in our patients, for frequently the symptom of pain is present only in the earlier stages of disease, and ceases entirely when the caries has advanced beyond the enamel, and if it is not arrested before this feeling of discomfort has passed away, the attention of the patient may not be directed to it again, unless he is in the habit of thus observing his teeth, until the near approach to the pulp warns him that he has a carious tooth.

As a rule, if the decay can not be removed with a cutting instrument or file, the tooth should be filled. It frequently requires more skill to fill a cavity thus early than when the decay is deeper seated, and sometimes we are tempted to send our patients away with the remark that the tooth has not decayed sufficiently to fill. Delay in such cases is dangerous. The patient may be prevented, unadvisedly perhaps, returning until the decay has progressed so far as to endanger the vitality of the tooth, and then there is more uncertainty as to the ultimate success of the operation.

If, then, we have decided to fill the teeth, and the decay is upon the approximate surface, the teeth should be separated if practicable, or cut away sufficiently to insure an easy approach to the cavity, and in such a manner that when the teeth approximate each other again, they can readily be cleansed upon their cut surfaces.

Care should then be exercised to remove every portion of decayed dentine, unless by doing so, we would expose the pulp. If this is liable to occur, sufficient of the softened bone can ordinarily be left as a protection to the parts beneath. The borders of the cavity, if the decay is deep-seated, and has rendered them thin and friable, should be cut down until they are strong, if possible, and smooth. Great care is required in the preparation of the cavity. It must not only be of a form to retain the filling, but should be shaped so that every portion of the material introduced for the plug may be thoroughly consolidated. Considerable care is required in shaping the cavity, lest too much be cut away from portions of the cavity already weak from the extent of the decay, rendering them more liable to break under the pressure necessary to consolidate the plug.

It is requisite that the cavity should be kept perfectly dry while the filling is being introduced, the material for which should be the best of its kind, and carefully prepared. Lastly, the finishing of the plug must receive due attention, for the ultimate success of the operation depends greatly upon the amount of care bestowed upon this part of the operation. The plug may be perfect up to this point, but unless the overlapping portions of the filling are removed, and the surface properly finished, we have no assurance that the filling will accomplish, in every respect, what we intended it should.

We have thus briefly referred to several of the successive steps necessary to insure a successful result in filling teeth, without attempting to describe the various methods by which they are performed. These will receive due attention, I presume, in the course of remarks by the members on the subject.

